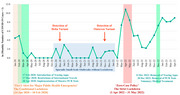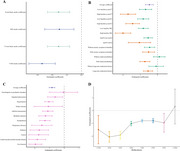# The Impact of COVID‐19 Pandemic on Cognitive Decline Among Community‐Dwelling Older Adults in Shanghai: A Longitudinal Study from 2009 to 2024

**DOI:** 10.1002/alz70860_105080

**Published:** 2025-12-23

**Authors:** Xiaowen Zhou, Hanzhi Deng, Qianhua Zhao, Ding Ding

**Affiliations:** ^1^ Institute of Neurology, Huashan Hospital, Fudan University, Shanghai, China; ^2^ National Center for Neurological Disorders, Huashan Hospital, Fudan University, Shanghai, China; ^3^ National Clinical Research Center for Aging and Medicine, Huashan Hospital, Fudan University, Shanghai, China; ^4^ Institute for Advanced Study in Social Sciences, Fudan University, Shanghai, China

## Abstract

**Background:**

The COVID‐19 pandemic has posed significant threats to health, with studies indicating worse cognitive performance and a higher risk of cognitive decline in community‐dwelling older adults following infection. Shanghai's abrupt and strict lockdown in April‐May 2022, namely a complete stay‐at‐home order. This period offered a unique opportunity to study the impact of COVID‐19 pandemic on cognitive decline in community‐dwelling older adults.

**Method:**

The Shanghai Aging Study is a community‐based longitudinal cohort study conducted in the Jingansi community of Shanghai. At baseline, 3792 residents aged≥50 years were enrolled between 2009‐2012. Demographic, medical histories, and anxiety symptoms were collected via face‐to‐face questionnaires, with APOE genotyping assessed through the TaqMan method. Baseline plasma *p*‐tau217, *p*‐tau181, and NfL were measured through a single‐molecule immune‐array assay. Follow‐up interviews were conducted between 2014‐2024, with cognitive function evaluated by the Mini‐Mental State Examination (MMSE) and domain‐specific cognitive tests. Studied period was divided into three waves: Baseline (2009‐2012, pre‐pandemic), Wave2 (Jan 2014‐Jan 2022, pre‐pandemic), and Wave3 (Jun 2022‐Dec 2024, post‐pandemic). The event‐study approach, the difference‐in‐differences approach and mixed linear effects model accounting for individual random effects were used to evaluate the pandemic's effect on cognitive decline and the rate of cognitive decline.

**Result:**

We identified steeper MMSE score declines with age in post‐pandemic years (2022‐2024) compared to earlier periods. An event‐study model adjusting for age, sex, and education, revealed a significant MMSE score decline in Wave3 (post‐pandemic) participants (β[95%CI]: ‐0.469[‐0.697, ‐0.242]) compared to baseline, while Wave2 (pre‐pandemic) showed no significant change. The decline was more pronounced in earlier birth cohorts, those with high baseline plasma *p*‐tau217, *p*‐tau181, NfL, APOE4 carriers, multi‐comorbidities, and long‐term medication use. The difference‐in‐differences model showed more significant declines in MMSE and executive function from Wave2 to Wave3(coefficient[95%CI]: ‐0.808[‐1.079, ‐0.537]). Mixed linear effects models further confirmed faster cognitive declines in both MMSE and executive function among pandemic‐exposed older adults. These results were valid when excluding participants dead before the pandemic.

**Conclusion:**

Our findings suggest that the COVID‐19 pandemic significantly accelerated cognitive decline in community‐dwelling older adults in Shanghai, and those with pre‐existing neurodegenerative conditions or mental health issues were particularly vulnerable.